# Gold and ZnO-Based Metal-Semiconductor Network for Highly Sensitive Room-Temperature Gas Sensing

**DOI:** 10.3390/s19183815

**Published:** 2019-09-04

**Authors:** Renyun Zhang, Magnus Hummelgård, Joel Ljunggren, Håkan Olin

**Affiliations:** 1 Department of Natural Science, Mid Sweden University, SE-851 70 Sundsvall, Sweden; 2 Department of Chemical Engineering, Mid Sweden University, SE-851 70 Sundsvall, Sweden

**Keywords:** metal-semiconductor network, gold particles, ZnO nanowires, gas sensors, room temperature sensors

## Abstract

Metal-semiconductor junctions and interfaces have been studied for many years due to their importance in applications such as semiconductor electronics and solar cells. However, semiconductor-metal networks are less studied because there is a lack of effective methods to fabricate such structures. Here, we report a novel Au–ZnO-based metal-semiconductor (M-S)_n_ network in which ZnO nanowires were grown horizontally on gold particles and extended to reach the neighboring particles, forming an (M-S)_n_ network. The (M-S)_n_ network was further used as a gas sensor for sensing ethanol and acetone gases. The results show that the (M-S)_n_ network is sensitive to ethanol (28.1 ppm) and acetone (22.3 ppm) gases and has the capacity to recognize the two gases based on differences in the saturation time. This study provides a method for producing a new type of metal-semiconductor network structure and demonstrates its application in gas sensing.

## 1. Introduction

The metal-semiconductor (M-S) structure [1,2] is one of the most important structures in semiconductor devices such as photodetectors [3]. Because of its importance, M-S interfaces [4], junctions [5], and hybrid structures [6] have attracted attention in both theoretical simulations and experiments.

M-S hybrid structures have different construction modes. One of the typical structures is a metal layer deposited on a semiconductor surface [7]. Such a planar structure has been used in many semiconductor devices, such as solar cells [4]. Recently, a novel metal-semiconductor-metal (M-S-M) structure was constructed on a single nanowire [8] and 2D materials such as grapheme [9] and MoS_2_ [10] to create micro-sized semiconductor devices.

In addition to M-S and M-S-M structures, there is another M-S network [(M-S)_n_] structure in which semiconductor nanowires are connected by a metal particle-forming network. However, such a structure is less studied because of the lack of an effective and efficient method to fabricate it.

From a materials point of view, ZnO is one of the most studied semiconductor materials in the M-S and M-S-M structures because of its unique electrical, optical and photochemical properties. Another reason is that the structure of ZnO, especially the nanostructures, can easily be tuned for specific needs using either chemical or physical methods. The other material—the metal in the M-S and M-S-M structures—can vary depending on the application. The most studied metal is gold because it is an inert material and has low contact resistance with semiconductors.

In this paper, we report a simple method for producing an (M-S)_n_ network by growing ZnO on a gold particle matrix that produces a melting gold nanowire network film [11,12,13,14]. Mechanisms of the network forming process have been suggested based on the topographical structure of the gold particles. A highly sensitive gas sensor was fabricated based on the (M-S)_n_ network to sense ethanol and acetone gases at room temperature.

## 2. Experimental Section

**Figure 1:**
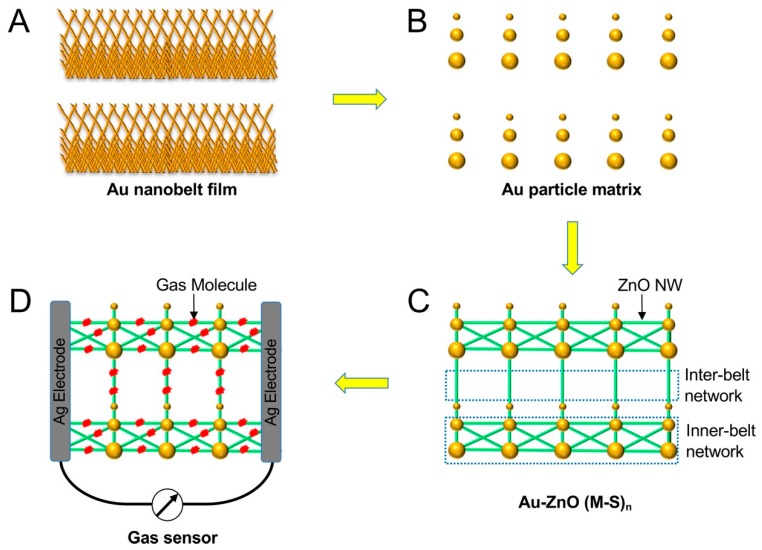
Schematic drawing of the fabrication processes of the Au–ZnO (M-S)_n_ network and the construction of a gas sensor.

Figure 1 shows a schematic drawing of this study, indicating the growth of the Au–ZnO (M-S)_n_ network and the gas sensing. The first step is to deposit a gold nanobelt film on a SiO_2_ wafer to grow the Au–ZnO (M-S)_n_ network. Such gold nanobelts were grown using an evaporation-induced self-assembly method that has been reported previously [11]. When the gold nanobelts were heated in the tube furnace during the physical vapor deposition (PVD) process, the gold melted, and gold particles with sizes ranging from 50 to 1000 nm formed. Such gold particles catalyze the growth of ZnO nanowires (ZnO NW), leading to the formation of Au–ZnO (M-S)_n_ networks. A gas sensor was then fabricated by depositing two silver electrodes on the Au–ZnO (M-S)_n_ film. The resistance change of the Au–ZnO (M-S)_n_ film was measured when exposed to ethanol or acetone gases. Photographs of the deposited gold nanobelt film and the sensors are given in the Supplementary Materials (Figure S1).

### 2.1. Growth of a Gold Nanobelt Pattern

The gold nanobelt pattern was fabricated using evaporation-induced self-assembly, with the method reported in [11]. Briefly, 100 mL 1 wt% HAuCl_4_ solution was heated to 100 °C, followed by adding 6 mL 1 wt% citric acid, and the reaction was allowed to continue for 5 min. Then, the resulting solution was kept in a 100 mL beaker under ambient conditions to allow the gold nanoparticles to assemble in nanobelts on the wall of the beaker.

### 2.2. Growth of the (M-S)_n_ Network of Au–ZnO

After the gold nanobelts were grown on the beaker wall, distilled water was carefully added to the beaker so that the nanobelts could separate from the beaker wall and float on the water surface. Then, the nanobelt film was picked up by using a piece of silica wafer. The wafer was subsequently inserted into a PVD reactor to grow the (M-S)_n_ network. The growing process was performed at 914 °C and 9 mBar under the protection of argon gas. A mixture of graphite and ZnO powder (1:1) was used as the material source for growing ZnO nanowires.

To study the growth process, the reaction was stopped after 2, 15 and 20 min, and the sample was removed for further microscopic characterization.

### 2.3. Characterizations

The structures of the gold nanobelt, gold particle matrix, and (M-S)_n_ film were imaged using a ZEISS EVO 50 scanning electron microscope (SEM). The ZnO nanowires were also imaged with a JEOL 2000FX transmission electron microscope (TEM).

### 2.4. Gas Sensing

Two silver electrodes were deposited on the (M-S)_n_ film, creating a gas sensor that had a 1 × 2 mm^2^ Au–ZnO network film between the two electrodes. A micromanipulator 1800 wafer probe station (Micromanipulator) was connected to the two electrodes to measure the resistance changes. The gas sensing experiments were performed at 20 °C by two different approaches: (1) the sensor was placed inside a sealed box, and a certain amount of ethanol or acetone was injected into the box and allowed to evaporate; (2) nitrogen gas flow was first injected into liquid ethanol or acetone and then blown over the (M-S)_n_ network films. For the gas sensing in a sealed box, different amounts of ethanol (99.5%) and acetone (99.5%) were injected into the box and equilibrated naturally at room temperature. The resistance of the Au–ZnO network was measured 30 min after the injection; hence, the liquids were completely evaporated in the air, especially when a large amount of liquid was injected into the box.

## 3. Results and Discussion

### 3.1. Growth of the Au–ZnO (M-S)_n_ Film on SiO_2_ Wafer

**Figure 2:**
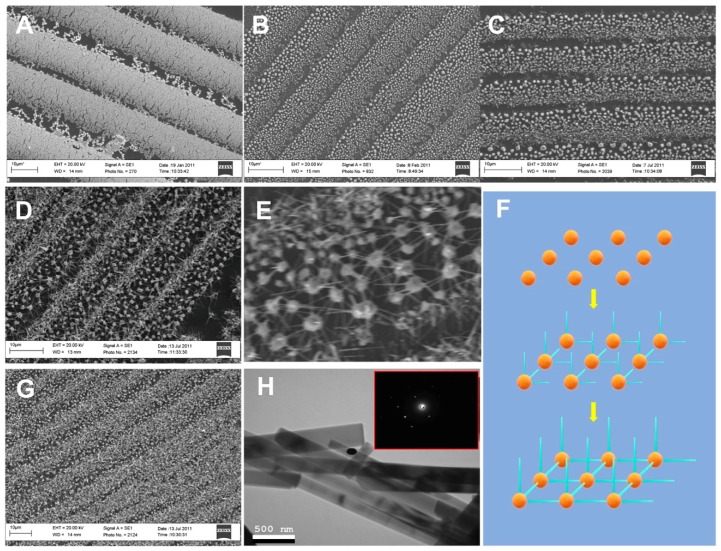
(**A**) Scanning electron microscope (SEM) image of gold nanobelts. (**B**) SEM image of gold nanobelts after annealing at 1100 °C. (**C**) SEM image of the Au–ZnO network after 2 min and (**D**) 15 min of growth. (**E**) Higher magnification of the Au–ZnO network. (**F**) SEM image of Au–ZnO (M-S)_n_ nanostructures grown at 20 min. (**G**) SEM images of Au–ZnO (M-S)_n_ nanostructures grown at 30 min. (**H**) Transmission electron microscope (TEM) images of ZnO nanowires from the Au–ZnO network. The insets show electron diffraction patterns. The black dots are imaging artifacts.

The PVD method was used to grow the Au–ZnO (M-S)_n_ network, and the process was investigated by imaging the samples after different growing times. Figure 2A,B show the SEM images of the gold nanobelt film and the gold particle matrix created by melting the gold nanobelt film. As the image shows, the density of the gold nanowires in the gold nanobelt film is not uniform (Figure 2A), which results in different sizes of gold particles (Figure 2B).

The ZnO nanowires grown on gold particles after 2, 15 and 20 min were imaged using SEM. After 2 min, the ZnO nanowires started to grow on small gold nanoparticles due to their stronger catalytic capabilities (Figure 2C). After 15 min, an Au–ZnO (M-S)_n_ network was observed (Figure 2D), and the horizontally-grown ZnO nanowires on large gold particles reached neighboring gold particles, forming network structures (Figure 2E). Figure 2F shows a schematic drawing of the growing process. After 20 min, more vertically-grown ZnO nanowires were observed (Figure 2G). TEM analysis of the ZnO nanowires (Figure 2H) indicated that the diameters were in the range of 50 to 200 nm.

**Figure 3:**
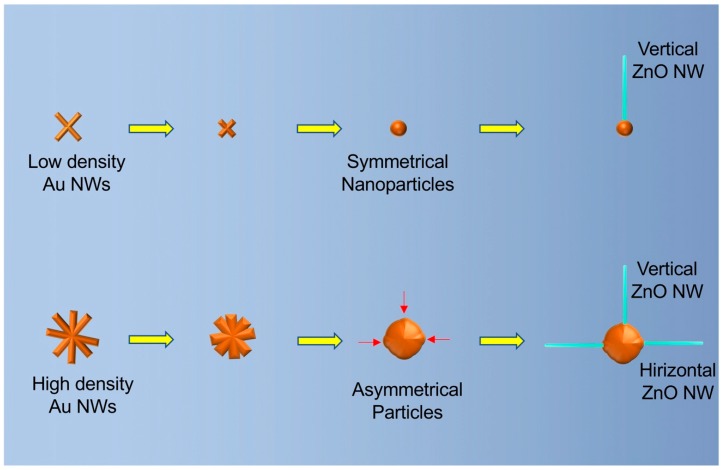
Schematic drawing of the mechanisms of the growing behaviors of ZnO nanowires on small (top) and large (bottom) gold particles. The red arrows indicate the high surface energy parts on the large gold particles.

The Au–ZnO (M-S)_n_ network was based on horizontally -rown ZnO nanowires that connect with neighboring gold particles. This behavior of growing horizontally is different from the reports in which the ZnO nanowires were vertically grown. We further studied the mechanisms behind the growth behavior of ZnO nanowires in our experiments based on the shape of the gold particles. Small gold nanoparticles formed in the region when the original density of gold nanowires is low (Figure 3A). Such gold nanoparticles are spherically shaped and have an even surface energy at the surface. Such a symmetrical shape only allows the ZnO nanowires to grow vertically. For the large gold particles (Figure 3B), their shapes are not symmetrical, and the surface energy is not even. Some parts have a higher surface energy than other parts, and that catalyzes the growth of the ZnO nanowires. The relative directions of the high surface energy parts to the center of the particle could be either vertical or horizontal, leading to the different growth directions of the ZnO nanowires.

### 3.2. Sensing Ethanol and Acetone Gases

ZnO nanowires have high sensitivity to gases such as ethanol and acetone at high temperature [15,16] due to their catalytic effect. However, the room-temperature sensing of such gases is less studied [17] because the catalytic effect is very weak at room temperature. In this work, we fabricated a room-temperature [18,19] ethanol and acetone sensor using the Au–ZnO network. The scheme of the sensor is shown in Figure 1D, where two silver electrodes were deposited on the network film, creating a gas sensor that has a 1 × 2 mm^2^ Au–ZnO network film between the two electrodes.

**Figure 4:**
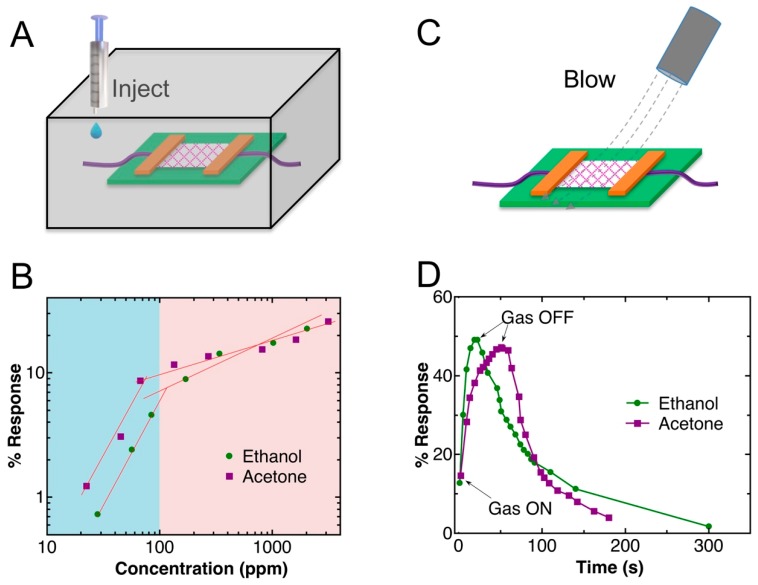
(**A**) A schematic drawing of the structure of gas sensing inside a sealed box. (**B**) Log–log plot of the responses of the sensor vs. the concentrations of the gases. (**C**) A schematic drawing of gas sensing under a flow of gases. (**D**) Gas response transients of the Au–ZnO (M-S)_n_ network.

Gas sensing on the fabricated sensor was first tested in a sealed box, as shown in Figure 4A, where different amounts of ethanol and acetone were injected into the box. Figure 4B shows the log–log plots of the response of the sensor to different concentrations of ethanol and acetone. For both gases, a linear region was observed at concentrations below 100 ppm, followed by another linear region. The linear region at low concentration has a higher sensitivity (greater slope), which is due to the absorption of the gas molecules on the ZnO nanowires, changing the Fermi level dramatically. However, the change is not significant at higher concentrations. The detection limits in our experiment were 28 ppm for ethanol and 22 ppm for acetone. Such detection limits are close to those of the state-of-the-art room-temperature ethanol and acetone sensors [20,21]. The concentration of the gases was calculated using Equation (1) [22]:

(1) $$C\left(ppm\right)=2.46\times \left(\frac{\rho V_{L}}{V_{B}M}\right)\times 10^{3}$$

where ρ (g/mL) is the density of the acetone or ethanol liquid, *V_L_* (µL) is the volume of liquid that was injected into the box, *V_B_* (L) is the volume of the box, and *M* (g/mol) is the molar weight of the molecule.

We also tested the response of the sensor under gas flow (Figure 4C), where ethanol and acetone gas flows were generated by bubbling the liquids with nitrogen. Nitrogen was selected because it has no effect on the resistance of the network. Figure 4D shows the real-time response of the sensor to the gas flows, where 50% and 47% changes in resistance were observed for ethanol and acetone, respectively, until the electric signal reached the steady stage. The response of the sensor to ethanol was found to be faster than that to acetone, which might be due to the faster binding process of ethanol molecules to the ZnO nanowires.

**Figure 5:**
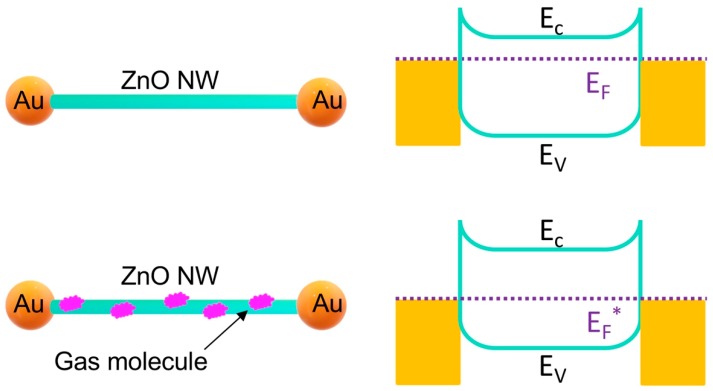
Mechanisms of room-temperature gas sensing based on the Au–ZnO (M-S)_n_ network.

The mechanism (Figure 5) behind the gas sensing is the decrease in the Fermi level of the ZnO nanowires due to the absorption of gas molecules, which changes the Fermi level at the contacts of gold and ZnO, leading to the increase of the electrical resistance. In the Au–ZnO (M-S)_n_ network, all ZnO nanowires that effectively contribute to the gas sensing are connected with two gold particles. Therefore, the summary of all the responses of the ZnO nanowires leads to a significant resistance change of the sensor and thus detection sensitivity. Such room-temperature gas sensing has a different mechanism from gas sensing at high temperature [18,23,24,25]. At high temperature, the reaction of gas molecules with the absorbed oxygen at the ZnO surface dominates the signal generation. At high temperature, the absorbed oxygen forms O^−^ [25], which can react with ethanol or acetone. At room temperature, the reaction of gas molecules occurs less because the absorbed oxygen is not in the form of O^−^; therefore, the signal change is considered mainly due to the absorption of the gas molecules on the ZnO nanowires, which leads to the change of the Fermi level, which is an important factor of the performance [23] of our sensor.

Compared to reported room-temperature sensors (Table 1), the detection limits of our Au–ZnO (M-S)_n_ network-based gas sensors for ethanol are close to the state-of-the-art values. The sensing of acetone is approximately 22 times higher than the reported one; the difference is that our experiment is performed in an atmosphere with a humidity of 35%, while it is 5% in the report. Such high humidity might lead to a weaker response.

## 4. Conclusions

In summary, we report a method of fabricating Au–ZnO metal-semiconductor networks by combing the evaporation-induced self-assembly method and the PVD method. Growth processes were imaged, and a possible mechanism has been suggested based on the size and the geometry of the gold particles. Furthermore, this Au–ZnO metal-semiconductor network has been used for the gas sensing of ethanol and acetone at room temperature, and the results indicated a high sensitivity gas sensor with detection limits of 28 ppm for ethanol and 22 ppm for acetone, which are close to the state-of-the-art values. 

## Figures and Tables

**Table 1:** Room-temperature ethanol and acetone gas sensors using ZnO nanomaterials.

Morphology	Gas	Concentration (ppm)	Response*	Response Time	Recovery Time	Ref.
Nanorods	Ethanol	200	111%	NA	NA	[26]
Nanorods	Ethanol	100	102%	45 s	50 s	[27]
Nanowires	Ethanol	20	110%	NA	15	[28]
Nanotubes	Ethanol	10	131%	NA	NA	[20]
(M-S)_n_ network	Ethanol	28	100.7%	19 s	280 s	This work
Thin film	Acetone	100	760%	34 s	40 s	[29]
Hierarchical	Acetone	1	102%	190 s	298 s	[21]
(M-S)_n_ network	Acetone	22	101%	51 s	130 s	This work

*The original response values in the articles have been converted to percentages.

## Supplementary Materials

The following are available online at https://www.mdpi.com/1424-8220/19/18/3815/s1, Figure S1: Photograph of gold nanobelt on silica wafer, silver electrodes deposited on (M-S)_n_ film, and the mounted sensor in a box.
