# Supplementary material for: Gold and ZnO-Based Metal-Semiconductor Network for Highly Sensitive Room-Temperature Gas Sensing
Source: Sensors (Basel). 2019 Sep 4;19(18):3815. doi: 10.3390/s19183815 (PMC6767169; doi:10.3390/s19183815)
Supplement: Supplementary file 1 [file sensors-19-03815-s001.pdf]

sensors

MDPI

Communication

Gold and ZnO-Based Metal-Semiconductor Network for Highly Sensitive Room-Temperature Gas Sensing

Renyun Zhang [1,*], Magnus Hummelgård [1], Joel Ljunggren [2] and Håkan Olin[1]

[1] Department of Natural Science, Mid Sweden University, SE-851 70 Sundsvall, Sweden
[2] Department of Chemical Engineering, Mid Sweden University, SE-851 70 Sundsvall, Sweden
* Correspondence: renyun.zhang@miun.se

Received: 9 August 2019; Accepted: 3 September 2019; Published:

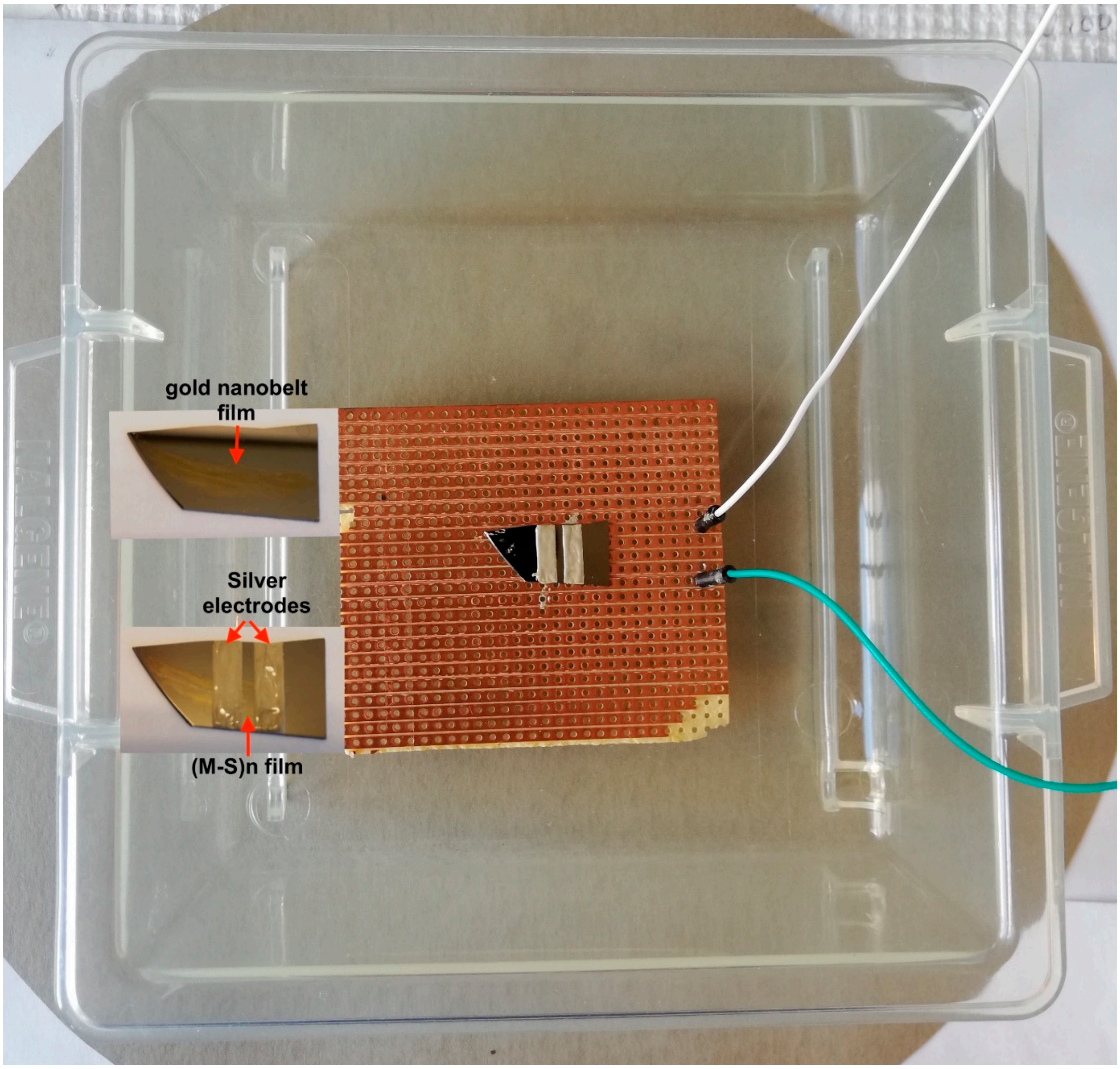

Figure 1. Photograph of gold nanobelt on silica wafer, silver electrodes deposited on $(M\text{-}S)_n$ film, and the mounted sensor in a box.
